# Guidance provided by pharmacists to customers regarding to destination of unused household medications: disposal of household medications

**DOI:** 10.1186/s12913-023-10319-8

**Published:** 2023-12-04

**Authors:** Aparecida de Fatima Michelin, Neuza Alves Bonifácio, Walter Bertequini Nagata, Valéria Maria Savoya da Silva, Laura Emilia Michelin Gobbo, Katia Denise Saraiva Bresciani

**Affiliations:** 1 School of Veterinary Medicine, São Paulo State University (Unesp), Rua Clóvis Pestana, no 793, Araçatuba, 16050-680 SP Brazil; 2 Institute of Health Sciences, Paulista University, Araçatuba, SP Brazil; 3 Secretary of Agriculture and Supply, Coordination of Agricultural Defense, Lins, SP Brazil; 4 Faculty of Medicine, San Camilo University Center, São Paulo, SP Brazil

**Keywords:** Attitudes, Practice, Disposal, Unused medicines, Household medication, Phamacists, Brazil

## Abstract

**Background:**

Discarding pharmaceuticals in the garbage or into the sewage system are still the most common methods in many countries. This study aims to investigate the guidance provided by pharmacists to customers on the disposal of unused and expired household medications in São Paulo State, Brazil.

**Method:**

The study population consisted of 630 pharmacists from the State of São Paulo, who work in community pharmacies. They answered an online questionnaire with questions composed in three blocks: demographic, work, and academic information on the pharmacist; guidance about the disposal of household medications; and knowledge regarding the reverse logistics of these medications. An invitation to participate in the questionnaire was made via WhatsApp, individually and collectively. Inferential statistics were performed using the chi-square test and were considered significant when *p* < 0.05%.

**Results:**

Among the participating pharmacists, the majority were women under 60 years old,56 (8.89%) stated that they never orient the customer regarding the disposal of unused and expired household medications, while 574 (91,12%) indicated that they almost provide guidance. The frequency with which they provided guidance was influenced by the number of years since graduation (*p* = 0.0047), the time they had worked in pharmacies and drugstores (*p* = 0.0007), and whether or not they had a graduate degree (*p* = 0.0181). Regarding the disposal of medications, among the 643 responses provided by the pharmacists,516 (80.25%) indicated that they oriented customers to return them to a pharmacy.

**Conclusion:**

A small number of pharmacists always orient customers on the proper disposal that should be followed for unused and expired household medications, prioritizing their return to a pharmacy. In general, these pharmacists have longer periods of work experience and higher academic qualifications. Thus, it is important to increase knowledge through professional training and further education programs.

**Supplementary Information:**

The online version contains supplementary material available at 10.1186/s12913-023-10319-8.

## Background

The proper disposal of unused medications, including those that have expired, has become a serious public health problem. Discarding pharmaceuticals in the garbage or in to the sewage system are still the most common methods in many countries, especially where there are no regulations regarding this procedure [1]. This practice results in the presence of active pharmaceutical compounds in the ecosystem, culminating in actions pathogenic to human, animal and environmental health [2]. This is evidence of the need to promote educational actions on the proper disposal of these wastes, rather than educating people on the risks associated with pharmaceuticals [3].

Numerous antibiotics have already been detected in water of lakes and rivers, constituting a major global threat, particularly in urban centers with a high population density [4, 5]. The easy migration of antibiotics in drinking water causes serious microbial resistance to these drugs, and entails environmental risks due to residual release into the ecosystem [6, 7]. Water and soil contamination has a vital influence on the structure of the ecosystem, and the main sources of pollution by antibiotics are the pharmaceutical industries, animal waste from livestock, and human waste from hospitals and domestic activities [8].

In the current era of growth in the pharmaceutical industry, the adoption of legislation to monitor and control the disposal of unused and expired medications is urgently required. However, there is no comprehensive global approach, and while general recommendations are supported by global regulatory bodies, treatment and implementation differ according to each country [9].

The worst effects of waste disposal on the environment likely occur in countries where landfills and open-air garbage dumps predominate and are not properly regulated. In both cases, contamination of the aqueous medium, both surface and groundwater, by medications is inevitable [1]. Burning waste, a practice adopted in many countries, is also not a wholly ecological method, given that most pharmaceutical compounds are organic substances that require sufficient oxygen, time and temperature for complete incineration [10].

In Brazil, unused and expired commercial and custom formulation medications used at home must be returned to their origin through a process of reverse logistics, together with their packaging [11]. In the State of São Paulo, a commitment was signed to comply with and fulfill this objective [12]. However, while we have observed the beginning of dealing with this problematic, it requires the joint effort of the authorities, the consumers, and the general public in the entire country to avoid environmental pollution by medications so we can achieve an ecologically balanced environment and a sustainable society [13].

Patients must be educated to dispose of medicines in a safe and appropriate way [14] and the pharmacist, along with other health professionals, must be prepared to assume the relevant role of guiding and educating the population to correctly dispose of unused medicines [15]. However, there is a concern regarding the exercise of this practice by pharmacists in community pharmacies, since the guidelines provided are not always adequate, such as, for example, disposing of medicines in the trash [16]. Most pharmacists do not advise patients on drug disposal and many of them discard expired drugs in the trash at their workplace [17].

Diagnosing the situation regarding the disposal of medications for use at home is necessary to provide a set of guidelines to implement a program for the management of these wastes. Therefore, the purpose of this study was to obtain information on the guidance given to customers by pharmacists about the disposal of unused and expired household medications.

## Methods

### Study design

This was a descriptive, cross-sectional survey, conducted through prevalidated electronic questionnaire (Google Forms)on the disposal of medications purchased at pharmacies, which had not been used or whose validity had expired. The study was conducted in the State of São Paulo, Brazil, between January to February 2022.

### Study population

630 pharmacists registered at The Regional Pharmacy Council of São Paulo State (RCP-SP) and who worked at community pharmacies in the State participated in this study. 74.621 pharmacists were registered at The Regional Pharmacy Council of São.

Paulo State, and it estimates that around 50.000 acted at these pharmaceutical establishments (CRF-SP, 2022). The invitation to participate in this study was sent by pharmaceutical leaders from the 27 regional units of São Paulo State to 6.750 pharmacists joined in WhatsApp groups. 630 pharmacists accepted to participate in the study, who signed the Informed Consent Form and answered the survey.

### Study instrument and data collection

The data collection instrument was adopted and modified from previously published studies by Bashaar et al. (2017) [18] and Wang et al. (2021) [19]. This instrument was composed by objective questions, containing independent variables, such as: pharmacists’ demographic, professional, and academic information. The dependent variable was the type of guidance provided by the pharmacist regarding disposal of household medications. The following options for guidance on the disposal of medicines were presented to pharmacists: household waste, sewage system, surface water, soil and return to a pharmacy.

Certain questions allowed more than one answer. The questionnaire was reviewed by experts and pretested on 12 respondents. No changes were necessary and pilot questionnaires were included in the sample. Thus, those who chose to were able to respond in a timely manner, within the 60-day period of availability, after which the form was closed after seven days without receiving responses. Therefore, a convenience sample was considered.

### Data analysis

Inferential statistics were performed, using the chi-square test to verify the association between the fact that the pharmacist provided guidance to the customer on the disposal of unused and expired household medications and the variables of interest, including: “How long is it since you undergraduate degree?”; “How long have you been working in a pharmaceutical establishment (pharmacy or drugstore)?”; “Do you have a graduate degree?”; and “Are you aware of Brazilian legislation on the reverse logistics of household medications?”. The statistical results were considered significant when *p* < 0.05%.

### Ethical considerations

The study was performed in accordance with the Declaration of Helsinki and approved by the Research Ethics Committee of the Araçatuba School of Dentistry (FOA) of São Paulo State University (UNESP), in accordance with report no.4.854.162. Each pharmacist signed a term of free, informed consent to participate in this research and the researchers guaranteed the anonymity and confidentiality of the responses.

## Results

The study population was 630 pharmacists who work in community pharmacies. Among these, 482 (76.51%) were women, 148 (23.49%) were men, and 612 (97.14%) were aged between 20 and 59 years old.

Among the participating pharmacists, 56 (8.89%) stated that they never orient the customer regarding the disposal of unused and expired household medications, while 574 (91,12%) indicated that they almost provide guidance. The results show that pharmacists who had finished undergraduate degree in pharmacy for a longer period (*p* = 0.0047), who had more extensive working experience in pharmacy (*p* = 0.0007), who had a graduate degree (*p* = 0.0181), and who knew about the reverse logistics of household medications (*p* < 0.001), more frequently advised customers about the disposal of these unused medications (Table 1).

**Table 1:** Guidance on the disposal of medications: professional and academic profile and knowledge of the pharmacists

Variable	Category	Do you provide guidance on the disposal of household medication to users/consumers?					P*
		Never	Rarely	Almost always	Always	Total	
How long you finished your undegraduate degree?	Less than 5 years	25	72	47	41	185	0.0047 ^1^
	From 6 to 10 years	12	64	52	35	163	
	More than 10 years	19	80	100	83	282	
	**Total**	**56**	**216**	**199**	**159**	**630**	
How long have you worked in a pharmaceutical establishment (pharmacy or drugstore)?	Less than 5 years	26	70	48	35	179	0.0007 ^1^
	From 6 to 10 years	16	59	54	35	164	
	More than 10 years	14	87	97	89	287	
	**Total**	**56**	**216**	**199**	**159**	**630**	
Do you have a graduate degree?	No	36	133	112	74	355	0.0181 ^1^
	Yes	20	83	87	85	275	
	**Total**	**56**	**216**	**199**	**159**	**630**	
Are you aware of Brazilian legislation on the reverse logistics of household medications?	No	34	123	74	34	265	> 0.0001 ^1^
	Yes	22	93	125	125	365	
	**Total**	**56**	**216**	**199**	**159**	**630**	

^1^Chi-squared test

Regarding the disposal of unused and expired medications, among the 643 responses provided by the pharmacists, 516 (80.25%) indicated that they oriented customers to return them to a pharmacy; moreover,430 (68.25%) pharmacists stated that the establishments where they work accepted these medications. However, 504 (80.0%) pharmacists stated that customers rarely or never ask for guidance on the disposal of these medications. When asked which professional should provide this guidance,571of the 963 (59.29%) responses provided indicated that this was the task of pharmacists (Table 2).

**Table 2:** Guidance on the disposal of unused and expired medications

Question	Category	n (%)
How do you orient users/customers regarding the disposal of unused or expired household medications?	Dispose of in household waste	9 (1.40)
	Discard down the toilet	5 (0.78)
	Return to a pharmacy	516 (80.25)
	Other options	113 (17.57)
	**Total**	**643 (100)**
Does the establishment where you work ACCEPT unused or expired medications for disposal?	Yes	430 (68.25)
	No	200 (31.75)
	**Total**	**630 (100)**
Do users/customers ask you for guidance on the disposal of household medications?	Never	78 (12.38)
	Rarely	426 (67.62)
	Almost always	96 (15.24)
	Always	30 (4.76)
	**Total**	**630 (100)**
Which professional should orient customers on the disposal of unused or expired household medications?	Pharmacist	571 (59.29)
	Nurse	198 (50.56)
	Doctor	194 (20.15)
	**Total**	**963 (100)**

Regarding the legislation on reverse logistics of unused and expired household medications, 365 (57.94%) pharmacists stated that they were aware of it. However, of these, 213 (58.36%) had not received any training on the subject. As for the improper disposal of these medications, 609 (96.67%) pharmacists thought they could affect the environment and human and animal health (Table 3).

**Table 3:** Reverse logistics of unused and expired household medications

Question	Category	n (%)
Are you aware of Brazilian legislation on the reverse logistics of unused and expired household medications?	Yes	365 (57.94)
	No	265 (42.06)
	**Total**	**630 (100)**
Have you received training on reverse logistics for medications?	Yes	152 (41.64)
	No	213 (58.36)
	**Total**	**365 (100)**
Do you think that the improper disposal of unused and expired household medications can affect the environment and human and animal health?	Yes	609 (96.66)
	No	3 (0.48)
	Don’t know	18 (2.86)
	**Total**	**630 (100)**

## Discussion

The purpose of this study was to obtain information on the guidance given to customers by pharmacists about the disposal of unused and expired household medications. Firstly, we confirmed that only 25% of pharmacists always advised customers on the proper disposal of unused and expired household medications. Among the guidance provided, the predominant option was returning the medications to a pharmacy, rather than disposing of them in household waste, discarding them down the toilet, or other options. This is in line with information they also provided that most pharmacies are capable of receiving these medications. Pharmacists who have had a degree and worked in pharmacies for longer periods, and those who have a graduate degree, more frequently advised customers on the disposal of unused medications.

Although the majority of pharmacists stated that they provided guidance fairly regularly, their knowledge may be lacking and they may not be providing information to customers consistently [20]. Thus, to orient the community on the disposal of unused medications, it is important to increase knowledge through professional training and further education programs [18, 21].

The population lacks information on the proper disposal of unused and expired medications, like in Saudi Arabia [22] and Turkey, for example, which is a situation of concern since a significant portion of the population has unused medications in their homes, [23] including antibiotics [22]. In Brazil, the general public has been disposing of medicines in household waste containers and in other inappropriate ways, including flushing them down the toilet or sink [24], practices also reported in several other parts of the world [25].

The majority of pharmacists assumed responsibility for orienting the population with regard to the disposal of unused medications, though this task can also be exercised by doctors and nurses. It is important that both pharmacists and physicians provide detailed information on the disposal of unused medications [26, 27] and that the importance of avoiding the prescription of unnecessary medications or in undue quantities is emphasized among physicians [26]. Community pharmacists find themselves in a strategic position to educate patients about the disposal of medications [18], since they are at the forefront of dispensing medication, which favors the provision of adequate education and community awareness.

Nevertheless, it was verified that a study carried out in Karbala, Iraq, with 129 pharmacists, demonstrated that these professionals had unsatisfactory knowledge about methods on disposal of medications [16], equally evidenced in a study released in The United Arab Emirates, which more thanone-third of pharmacists discarded pharmaceutical dosage forms, including solid as well as liquid, in unauthorized places, such as sinks and toilet seats [28]. Regarding to patients counseling about ways of disposal, a study released in Trinidad with 400 pharmacists evidenced that more than one-third of pharmacists don’t advise patients concerning the medication disposal [17], in this same perspective, a study carried out in South Africa, which the minority of healthcare professionals, including pharmacists, advise patients to adopt safe disposal practices, and, even more worrying is the majority of these professionals considering the incineration and discard in sinks and toilet seats as correct ways of medication disposal [15]. This practice was verified in Kosovo, as well, once more than a half of pharmacystudents asserted that discard expired and unused medications in the garbage [29].

The presented scenarios, herewith results of undertaken studies with pharmacists and pharmacy students, indicate that the first educational measures must be introduced in pharmacy courses program, in order to prepare future pharmacists with competences, abilities and necessary attitudes to adopt safe methods of medication disposal, as patients must be educated by these professionals for the purpose of avoiding accumulated medication and dispose medications in a safe and appropriate way, as stated by SammutBartolo, Azzopardi, Serracino-Inglott, (2021) [14].

Furthermore, continuing education programs must be implemented and adopted by pharmacists in order to reinforce awareness and knowledge regarding the disposal and safe destination of expired and unused household medications, as they can be prepared to guide patients and the population about the importance of disposing these products appropriately, as well as evidencing the negative impacts causable in biological ecosystems, people’s and animals’ safety.

The majority of pharmacists stated that customers rarely or never asked for guidance on the disposal of unused medications. This disinterest may reflect a lack of awareness of the risks arising from the inappropriate disposal of these products. The disposal of unwanted pharmaceutical products in environmentally unsafe ways and the lack of awareness of the impact on the ecosystem constitutes a warning that the adoption of strategies that strengthen the management of these wastes is required [30].

Efforts directed at individuals to raise awareness on proper disposal can be more effective than broad campaigns that pursue the same aim [31], so pharmacists should not miss the opportunity for an individualized approach with regard to the disposal of unused and expired medications, when dispensing medication and at other times when they provide pharmaceutical assistance. Raising people’s awareness of the negative effects on the environment and on public health, as well as the consequences of accidental exposure to medications through inappropriate disposal, will pave the way for the development and implementation of public policies aimed at the proper use and disposal of household medications that will enable society to control and reduce the impact of these effects [32–34].

It is worth noting that only slightly more than half of the pharmacists knew about Brazilian legislation on the reverse logistics of unused household medications, and less than half have received training on how to execute this practice, which is being gradually implemented in Brazil, though more rapidly in the State of São Paulo. This reality forces us to reflect on the probable inadequacies of didactic contents of undergraduate pharmacy courses on themes related to the environment, the lack of engagement by businesses with regard to public policies, together with disinterest or lack of opportunity on the part of pharmacists to access sources of information on this subject. Thus, we recommend that the curriculums of all health professionals are strengthened in terms of medication management, seeking innovative ways to approach the subject [15]. Health professionals play a crucial role in educating and orienting the general public in relation to consumer practices with regard to the disposal of unused and expired medications [35], such that without their effective participation, public policies aimed at the disposal of these wastes may not present the expected satisfactory results.

Since the majority of the pharmacists stated that the improper disposal of unused and expired household medications can affect the environment and human and animal health, public education programs and the development of facilities for the disposal of medications in order to protect the environment and improve community health are desirable [36]. Coupled with this, we should not forget the importance of rational prescribing and optimal dispensing practices [19], given that when these are not practiced, it further contributes to the waste of medications at home.

Collaboration and coordination between stakeholders are essential for the development of a successful national collection plan [37], since has to be admitted that pharmaceuticals have unfortunately escaped their industrial and domestic boundaries and have reached the environment, as exemplified by the presence of fluoroquinolones in bodies of water, which causes a high risk for aquatic organisms [38].

Pharmaceutical products are currently considered one of the main environmental pollutants and are ubiquitous in water and soil. Although acute risk assessments show negligible danger to human health, the delayed effects of these pollutants, including bioaccumulation, effects amplified by drug interactions, the exacerbation of bacterial drug resistance and reduction in aquatic and land food production, can no longer be ignored [39]. The inappropriate disposal of medications in the home has contributed significantly to achieving this level of pollution.

Therefore, it is necessary to adopt an educational approach to the control of environmental contamination, in order to support people in affirming their choices in terms of health and contributing to risk reduction [40]. Preventing risks that are demonstrably linked to the quality of our living environments is usually the responsibility of the authorities, but individuals can also adopt precautionary practices [41]. However, awareness of pharmaceutical pollution in the environment and access to information concerning the proper disposal are significantly associated with participation in take-back programs [42, 43].

## Conclusion

In this Brazilian study, we have evidenced that a small portion of pharmacists has the habit of always orientating customers on the proper disposal of unused and expired household medications. In addition, the consumers rarely or never request information about it. Therefore, it is necessary that all pharmacists are qualified on the subject and adopt proactive attitudes to promote adequate information on the proper disposal of medicines.

The present study, conducted among pharmacists, will add to the investigative literature for the implementation of new policies regarding the disposal of unused medicines. However, with limitations, considering the number of participants.

### Electronic supplementary material

Below is the link to the electronic supplementary material.

Supplementary Material 1

## Data Availability

The analysis results generated in the study are presented in this published article. The research data set are available upon request from the corresponding author.

## Abbreviations

FOA: Araçatuba School of Dentistry
UNESP: São Paulo State University
