# Supplementary material for: Guidance provided by pharmacists to customers regarding to destination of unused household medications: disposal of household medications
Source: BMC Health Serv Res. 2023 Dec 4;23:1350. doi: 10.1186/s12913-023-10319-8 (PMC10694965; doi:10.1186/s12913-023-10319-8)
Supplement: Supplementary file 1 — Supplementary Material 1 [file 12913_2023_10319_MOESM1_ESM.docx]

**Data collection instrument**

You are invited to participate in this research, which objects obtain information about knowledge, practice and pharmacist’s attitude regarded to household medication disposal.

Personal data will not be disclosed and ensures [spontaneity](https://www.linguee.com.br/ingles-portugues/traducao/spontaneity.html) while answering the survey.

As accepting to participate, it is necessary to read and agree to the Informed Consent Form, and afterwards, answer the survey.

In advance, we appreciate your contribution to this study be carried out.

Include the Informed Consent Form as a link.

( ) I hereby certify that I have read the Informed Consent Form and I accept to participate in this research.

The estimated time to answer the form is the less than 10 minutes.

**QUESTIONS 1 TO 6 – DEMOGRAPHIC, PROFESSIONAL AND ACADEMIC INFORMATIONS**

**Question 1.** Which age group do you belong to?

0( ) 24 years old or under

1( ) 25 to 34 years old

2( ) 35 to 44 years old

3( ) 45 to 54 years old

4( ) 55 to 64 years old

5( ) 65 years old or over

**Question 2.** Which gender group do you belong to?

0( )Male

1( )Female

**Question 3**. How long is it since you undergraduate degree in pharmacy??

0( ) Less than 5 years

1( ) From 6 to 10 years

2( ) From 11 to 15 years

3( ) From 16 to 20 years

4( ) From 21 to 25 years

5( ) From 26 to 30 years

6( ) From 31 to 35 years

7( ) More than 35 years

**Question 4.**How long have you worked in a pharmaceutical establishment (pharmacy or drugstore)?

0( ) Less than 5 years

1( ) From 6 to 10 years

2( ) From 11 to 15 years

3( ) From 16 to 20 years

4( ) From 21 to 25 years

5( ) From 26 to 30 years

6( ) From 31 to 35 years

7( ) More than 35 years

**Question 5.**Do you have a graduate degree?

0( ) No

1( ) Yes (In affirmative case, answer the question 5.1)

**Question 5.1.** What is your graduate degree?

0( ) Graduate Diploma

1( ) Master’s Degree

2( ) PhD

**QUESTIONS 6 TO 8 – HOUSEHOLD MEDICATION DISPOSAL GUIDANCE**

**Question 6.** Do you orient users/customers regarding the disposal of household medications?

0( ) Never (In this case, answer the question 6.2)

1( ) Rarely (In affirmative case, answer the questions 6.1 and 6.2)

2( ) Almost always (In affirmative case, answer the questions 6.1 and 6.2)

3( ) Always (In affirmative case, answer the questions 6.1 and 6.2)

**Question 6.1**. How do you orient users/customers regarding the disposal of expired or unused household medications? (You can choose more than one option).

0 ( ) Dispose in a household waste.

1 ( ) Discard down in the toilet.

2 ( ) Dispose in the bathroom sink or kitchen.

3 ( ) Dispose in streams, rivers or lakes.

4 ( ) Bury in the soil

5 ( ) Incinerate

6 ( ) Return to a pharmacy

7( ) Other options (specify) ______________________________________________

**Question 6.2.** Which professional should orient customers on the disposal of expired or unused household medications? (You can choose more than one option).

0( ) Pharmacist

1( ) Nurse

2( ) Doctor

3( ) Other options (specify) ______________________________________________

**Question 7.** Do users/customers ask you for guidance on the disposal of household medications?

0( ) Never

1( ) Rarely

2( ) Almost always

3( ) Always

**Question 8.** Does the establishment where you work ACCEPT expired or unused medications for disposal?

0 ( ) No

1 ( ) Yes (In affirmative case, answer the questions 8.1 and 8.2)

**Question 8.1.** Does the establishment where you work RECEIVE expired or unused household medications for disposal from users/costumers?

0( ) Never

1( ) Rarely

2( ) Almost always

3( ) Always

**Question 8.2.** Does the unused or expired household medication for disposal need to be acquired at the establishment where you work?

0( ) No

1( ) Yes

**QUESTIONS 9 TO 10 – REVERSE LOGISTICS KNOWLEDGE**

**Question 9.** Are you aware of Brazilian legislation on the reverse logistics of household medications?

0( ) No

1( ) Yes (In affirmative case, answer the questions 9.1; 9.2 e 9.3)

**Question 9.1*.** Where did you be aware of Brazilian legislation on the reverse logistics of household medications? (You can choose more than option).

0( ) Undergraduate course

1( ) Graduate course

2( ) Continuing education course

3( ) Regional Pharmacy Council

4( ) Social Media

5( ) Other (specify)_______________________________________________

*Not approached.

**Question 9.2.** Have you received training on reverse logistics of expired or unused household medications?

0( ) No

1( ) Yes

**Question 9.3.** Have you ever participated in a guidance campaign to general public about reserve logistics of expired or unused household medications?

0( ) No

1( ) Yes

**Question 10.**Do you think that the improper disposal of household medications can affect the environment and human and animal health?

0( ) No

1( ) Yes (In affirmative case, answer the question 10.1)

3( ) Don’t know

**Question 10.1.** How does the improper disposal of household medications can affect the environment and human and animal health?

|  |
| --- |
